# Predicting Metabolic Syndrome Using the Random Forest Method

**DOI:** 10.1155/2015/581501

**Published:** 2015-07-28

**Authors:** Apilak Worachartcheewan, Watshara Shoombuatong, Phannee Pidetcha, Wuttichai Nopnithipat, Virapong Prachayasittikul, Chanin Nantasenamat

**Affiliations:** ^1^Center of Data Mining and Biomedical Informatics, Faculty of Medical Technology, Mahidol University, Bangkok 10700, Thailand; ^2^Department of Clinical Chemistry, Faculty of Medical Technology, Mahidol University, Bangkok 10700, Thailand; ^3^Excellence Service Center for Medical Technology and Quality Improvement, Faculty of Medical Technology, Mahidol University, Bangkok 10700, Thailand; ^4^Department of Clinical Microbiology and Applied Technology, Faculty of Medical Technology, Mahidol University, Bangkok 10700, Thailand

## Abstract

*Aims*. This study proposes a computational method for determining the prevalence of metabolic syndrome (MS) and to predict its occurrence using the National Cholesterol Education Program Adult Treatment Panel III (NCEP ATP III) criteria. The Random Forest (RF) method is also applied to identify significant health parameters. *Materials and Methods*. We used data from 5,646 adults aged between 18–78 years residing in Bangkok who had received an annual health check-up in 2008. MS was identified using the NCEP ATP III criteria. The RF method was applied to predict the occurrence of MS and to identify important health parameters surrounding this disorder. *Results*. The overall prevalence of MS was 23.70% (34.32% for males and 17.74% for females). RF accuracy for predicting MS in an adult Thai population was 98.11%. Further, based on RF, triglyceride levels were the most important health parameter associated with MS. *Conclusion*. RF was shown to predict MS in an adult Thai population with an accuracy >98% and triglyceride levels were identified as the most informative variable associated with MS. Therefore, using RF to predict MS may be potentially beneficial in identifying MS status for preventing the development of diabetes mellitus and cardiovascular diseases.

## 1. Introduction

Metabolic syndrome (MS) is a complex disorder encompassing a cluster of metabolic abnormalities characterized by central obesity, hyperglycemia, hypertension, and dyslipidemia [1]. Particularly, progression of the pathophysiological state of MS is a consequence of the complex and interrelation of genetic and environmental factors including insulin resistance (IR), adiposity, dyslipidemia, endothelial dysfunction, elevated blood pressure, and chronic state [2]. In addition, MS is found to be associated with other abnormalities such as proinflammatory and prothrombotic states [2] while hematological parameters (i.e., white blood cell (WBC), red blood cell (RBC), hemoglobin (Hb), hematocrit (Hct), and platelet) have been shown to be correlated with IR and MS [3–7]. MS predisposes an individual to the development of diabetes mellitus (DM) and cardiovascular diseases (CVD) in which the prevalence is estimated to increase drastically to 360 million cases of DM by 2030 [8] and 20 million cases of CVD by 2015 [9]. Therefore, it is highly desirable to seek out ways for rapid identification of MS. The definition of MS emerged from collaborative efforts between many organizations such as the World Health Organization (WHO) [10], the European Group for the Study of Insulin Resistance (EGIR) [11], the National Cholesterol Education Program (NCEP) Adult Treatment Panel (ATP) III [12], and the International Diabetes Federation (IDF) [13].

The quantitative population-health relationship (QPHR) model is an approach for exploring the relationship between health parameters and the disease of interest. Machine learning techniques such as artificial neural network (ANN), support vector machine (SVM), decision tree (DT), and association rule analysis (AA) are employed to mine large amounts of data so as to discover unknown patterns [14] related to specific diseases. The QPHR approach has been shown to successfully predict and classify a number of diseases in clinical medicine such as MS [14–16], hypertension and hyperlipidemia [17], cancer [18], type 2 DM [19], cerebrovascular disease [20], and inflammatory bowel disease [21]. In the present study, an efficient ensemble-based method, Random Forest (RF), was used to predict the presence of MS, to determine its prevalence in an adult Thai population and to identify significant MS-associated health parameters. Particularly, such analyses were performed using physical (i.e., age, gender, WC, BMI, and BP) and biochemical (i.e., lipid profiles, FPG, and hematological indices) parameters.

## 2. Materials and Methods

### 2.1. Subjects

The data were obtained from 5,646 individuals (i.e., 2,028 men and 3,618 women) residing in urban areas in Thailand who received health check-ups from the Faculty of Medical Technology, Mahidol University, in 2008 [22]. Such data set is comprised of complete health parameters describing both physical and biochemical parameters. Individuals aged 18–78 years were characterized by measuring their health parameters, which encompassed (i) anthropometric testing such as waist circumference (WC), body mass index (BMI), and systolic/diastolic blood pressure (BP) (measured according to standard procedure) and (ii) blood testing including fasting plasma glucose (FPG), total cholesterol (CHOL), triglyceride (TG), low-density lipoprotein cholesterol (LDL-C), high-density lipoprotein cholesterol (HDL-C), white blood cell (WBC), hemoglobin (Hb), hematocrit (Hct), and platelet (PLT), all of which were analyzed at the Center of Medical Laboratory Services, Faculty of Medical Technology, Mahidol University. Blood samples taken after 12 hours of overnight fasting were subjected to standard enzymatic analysis using automated chemistry analyzers (Hitachi 911, Roche) for measuring the levels of the following biochemical parameters comprising CHOL, TG, HDL-C, LDL-C, and FPG. It is worthy to note that LDL-C was calculated according to Friedewald formula if TG is <400 mg/dL. Hematological parameters were determined using Hematology Analyzer (XT1800i, Sysmex). WC was obtained using a measuring tape while BMI was calculated as the ratio of weight (kg) to height (m^2^). Age (18–78 years old) was categorized into 5 groups comprising 18–24-, 25–34-, 35–44-, 45–54-, and ≥55-year-old groups to explore the prevalence of MS in an age-dependent manner.

### 2.2. Definition of MS

Individuals were defined as having MS according to NCEP ATP III criteria [12] using a modified WC cutoff for the Thai population [22]. Individuals with 3 or more of the following characteristics were classified as having MS: (i) central obesity by WC (≥87.75 cm for men and ≥80 cm for women); (ii) BP ≥130/85 mmHg or treatment of previously diagnosed hypertension; (iii) FPG ≥100 mg/dL or previously diagnosed type 2 diabetes; (iv) TG ≥150 mg/dL or specific treatment for triglyceride abnormality; and HDL-C <40 mg/dL in males or <50 mg/dL in females or specific treatment for an abnormal HDL-C.

### 2.3. Data Sampling

A data set was divided into 2 subsets by using principal component analysis (PCA) [23–25]. The first subset (i) was an internal test set or training dataset evaluated with a 10-fold cross-validation (10-fold CV) procedure. This data set was divided into 10 subsets of roughly the same size. During each 10-fold CV procedure, 9 subsets were used for training a predictive model, and the remaining subset was used for validation. Finally, the prediction result was obtained by averaging across the 10 cross-validation experiments. The second subset (ii) was an external test set or testing dataset that was used for evaluating the reliability of the predictive model.

To further validate the predictive performance, data splitting of the dataset was performed for 20 times followed by independent model construction. Afterwards, the mean and standard deviation of these 20 runs were computed for each statistical parameter.

### 2.4. Random Forest

Random Forest (RF) based on an ensemble-based decision tree [26, 27] is an extensively used ensemble learning method. Breiman and Cutler introduced the RF method to improve prediction performances of classification and regression trees (CART) by growing many weak CART trees [27]. To select feature importance, out-of-bag (OOB) data are used for evaluating feature importance as follows: (1) two-thirds of a training dataset is used to construct the predictive classifier and the remaining is used for evaluating the performance of such classifier and (2) the feature importance of each feature can be evaluated by measuring the decrease in prediction performance. The performance evaluation can be reported in terms of either accuracy or the Gini index. The Gini index is used to evaluate the ability of a potential discriminative of each feature that can be defined as 1 − ∑_*j*_ *p* ^2^(*j*∣*t*), where *p*(*j*∣*t*) is the estimated class probability for feature *t* or node *t* in a decision tree and *j* is an output data or class. In this study, *j* = 2 is represented as MS = Yes and MS = No. The mean decrease of the Gini index (MDGI) was used to select the important health parameters because MDGI is suggested to be more robust than the mean decrease of accuracy [28]. The health parameter with the largest value of MDGI is the most important feature because it contributes the most to the prediction performance. Decision rules were subsequently extracted from one of the representative decision trees from the Random Forest model.

### 2.5. Statistical Analysis

The statistical parameters for assessing the predictive performance of the RF classifier, accuracy (Acc), sensitivity (Sens), specificity (Spec), and Matthews correlation coefficient (MCC), were presented in the following equation [14]:

(1) Accuracy=TP+TNTP+TN+FP+FN×100,

(2) Sensitivity=TPTP+FN×100,

(3) Specificity=TNTN+FP×100,

(4) MCC=TP×TN−FP×FNTP+FPTP+FNTN+FPTN+FN,

where TP, TN, FP, and FN are the number of true positives, true negatives, false positives, and false negatives, respectively. An MCC coefficient of +1, 0, and −1 indicates a perfect prediction, no better than random prediction, and total disagreement between prediction and observation, respectively [29]. Statistical analysis was performed using Statistics 18.0 (SPSS Inc. USA) to compare differences between groups using an independent two-sample *t*-test with a *P* value less than 0.05 (<0.05) defined as statistically significant.

## 3. Results

### 3.1. Population Characteristics

**Figure 1:**
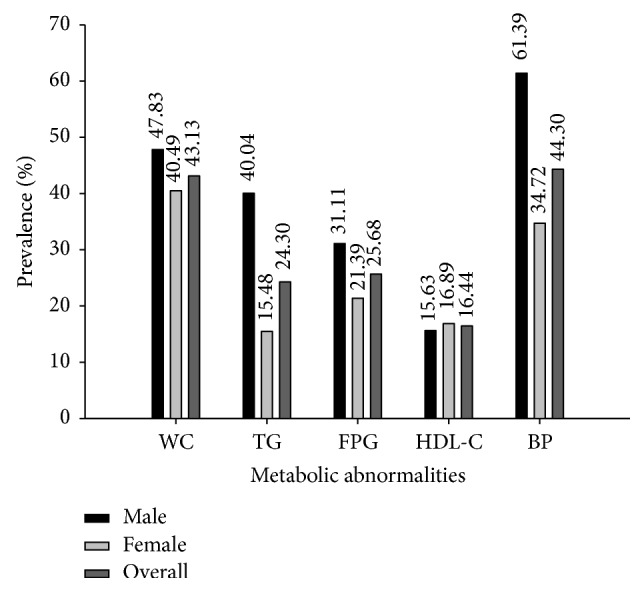
Individual components of metabolic syndrome in the study subjects.

The sample size was composed of 5,646 participants that included 3,618 (64.08%) women and 2,028 (35.92%) men. The prevalence of individual components of MS is displayed in Figure 1. Elevated BP (61.39%) was the most common metabolic abnormality in males followed by central obesity (47.83%), whereas central obesity (40.49%) was the most common metabolic abnormality in females followed by elevated BP (34.72%). Overall, elevated BP (44.30%) was the most common metabolic abnormality followed by central obesity (43.13%) in both males and females. In addition, the prevalence of low HDL-C was greater in females (16.89%) than in males (15.63%), whereas other metabolic abnormalities were greater in men than in women.

### 3.2. Prevalence of Metabolic Syndrome

**Figure 2:**
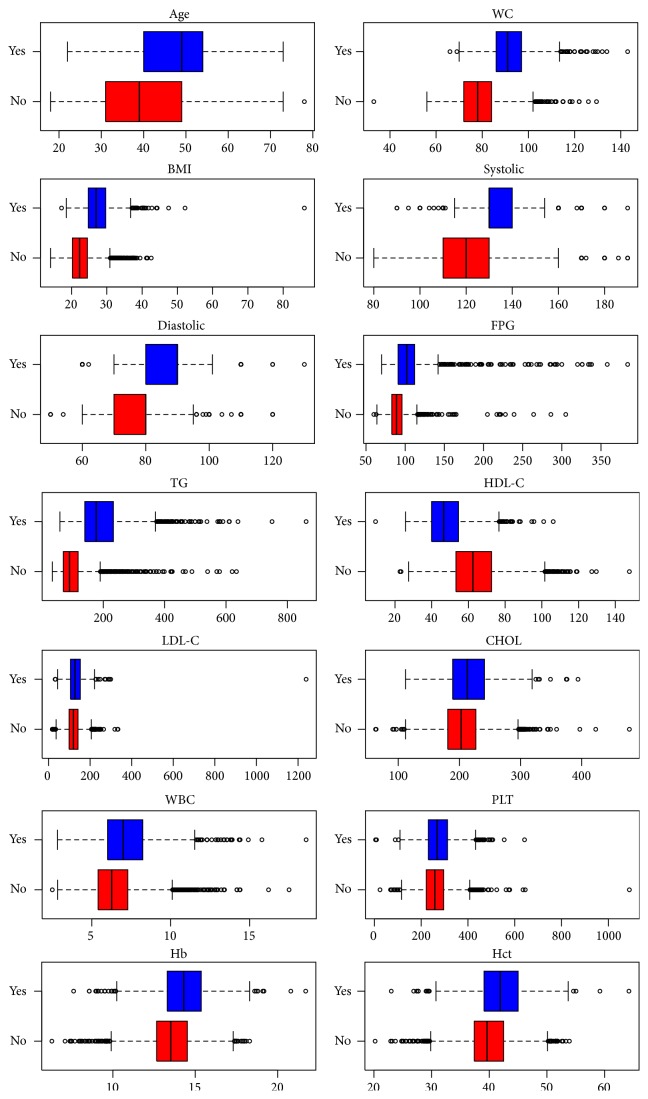
Box plots of biochemical parameters of metabolic syndrome (Yes) and nonmetabolic syndrome (No) groups.

**Figure 3:**
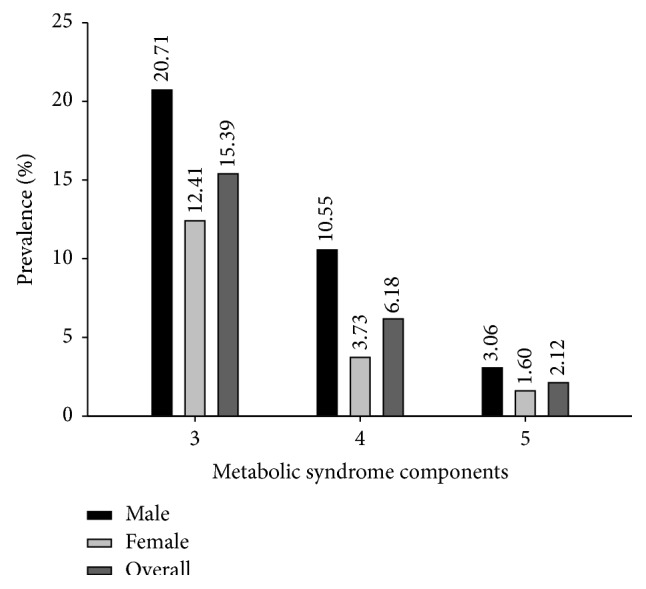
Prevalence of metabolic syndrome components among the subjects using NCEP ATP III.

**Figure 4:**
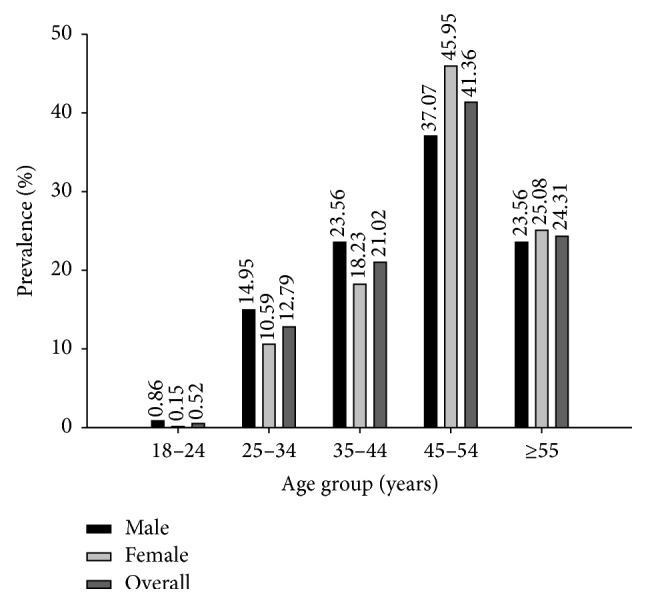
Prevalence of metabolic syndrome in different age groups.

The subjects were classified for MS using NCEP ATP III criteria composed of 3 or more metabolic components. Out of 5,646 individuals, 1,338 participants (642 females and 696 males) were identified as having MS and 4,308 participants (2,976 females and 1,332 males) as having non-MS.  Table 1 and Figure 2 display comparisons of the clinical and biochemical parameters of the MS and non-MS groups. The average value of all health parameters was higher in the MS than in non-MS group (*P* value < 0.001), except for HDL-C that was lower in MS than in non-MS group (*P* value < 0.001). The prevalence of MS was 23.70% using NCEP ATP III criteria and was higher in men than in women, 34.32% and 17.74% in males and females, respectively. Furthermore, the prevalence of 3 components of MS was higher than 4 and 5 components of MS and was more frequently observed in males (20.71%, 10.55%, and 3.06%, resp.) than in females (12.41%, 3.73%, and 1.60%, resp.) as shown in Figure 3. The overall prevalence of 3, 4, and 5 combination components of MS was 15.39%, 6.18%, and 2.12%, respectively. The common MS combinations of triplet and quartet metabolic components were WC + BP + TG and WC + BP + TG + FPG for males, respectively, and WC + BP + FPG and WC + BP + TG + HDL-C for females (data not shown). The prevalence of MS in males and females stratified by age is displayed in Figure 4. The prevalence of MS was age-dependent ranging from 0.86%–37.07% for men, 0.15%–45.95% for women, and 0.52%–41.36% for the total population. Interestingly, the prevalence of MS in the 18–24-, 25–34-, and 35–44-year-old groups was higher in males (0.86%, 14.95%, and 23.56%, resp.) than in females (0.15%, 10.59%, and 18.23%, resp.), while women in the 45–54- and ≥55-year-old groups had a higher prevalence of MS (45.95% and 25.08%, resp.) than men in the same age groups (37.07% and 23.56%, resp.). Overall, the prevalence of MS was highest in individuals who were 45–54 years old (41.36%) followed by those ≥55 years old (24.31%) and was lowest in the 18–24-year-old (0.52%) group.

### 3.3. Prediction of MS

In this study, the original dataset was composed of 5,646 participants. We excluded three individuals who did not have the following laboratory results: WBC, Hb, Hct, and PLT. The remaining dataset consisted of 5,643 participants. This data set was randomly divided into approximately 4,796 participants or 85% of 5,643 participants for an internal test set (10-fold CV) and approximately 847 participants or 15% of 5,643 participants for an external test set as displayed in Table 2. In constructing RF models, the number of trees (*n*_tree_) was varied from 10 to 50 (*n*_tree_ = 10, 20, 30, 40, and 50) and the number of selected features was set to the default value of the square root of the total number of features. The number of decision trees was selected from the predictive performance of RF providing the highest four measurements. The performance comparison among the various numbers of trees is shown in Table 3.

The statistical results of the internal test set at 10-fold CV using RF with *n*_tree_ = 40 were 98.02% accuracy, 94.81% sensitivity, 99.02% specificity, and 0.94 MCC, as calculated using (1)–(4), respectively. Interestingly, the simple RF with *n*_tree_ = 20 achieved the optimum prediction result for the external test set with 98.11% accuracy, 94.00% sensitivity, 99.38% specificity, and 0.95 MCC. Conversely, when a number of decision trees increased to 30, 40, and 50, their accuracy decreased to 97.76%. These results demonstrated the superiority of RF with *n*_tree_ = 20.

**Figure 5:**
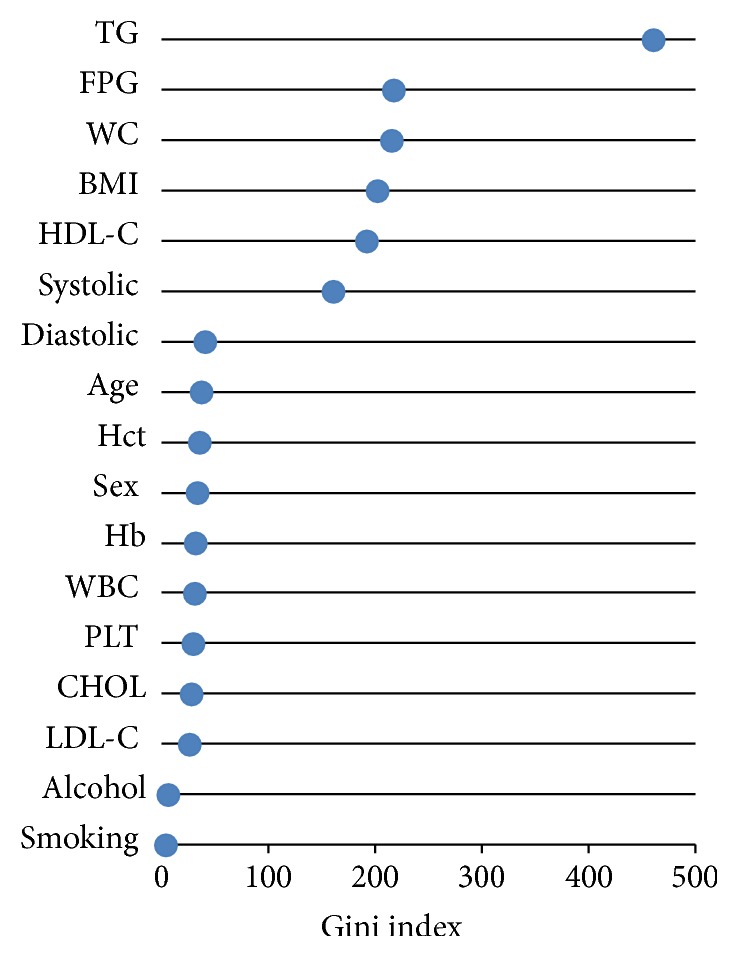
Health parameters importance graph.

**Figure 6:**
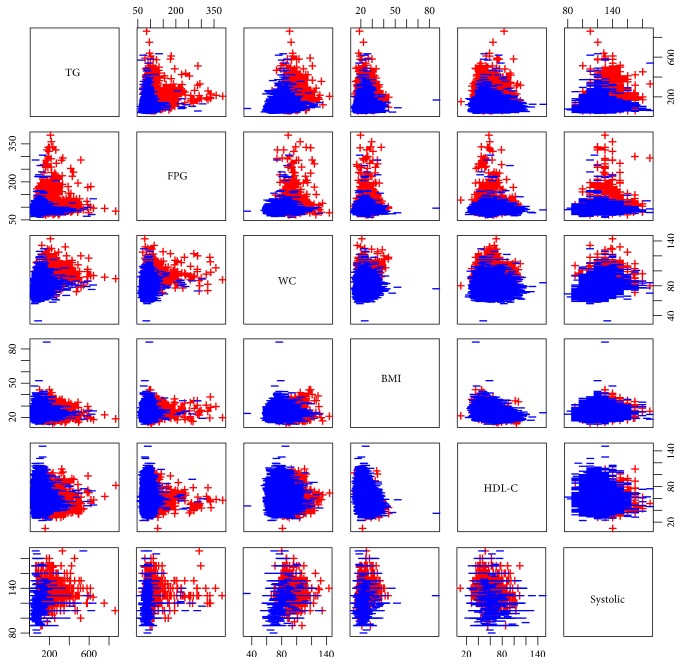
Scatter plots of MS component classifications: MS (red) and non-MS (blue) groups.

The discovery of essential health parameters was performed as showed in Figure 5. The parameter with the largest value of MDGI was considered to be the most important. The four top-ranked informative health parameters were TG, FPG, WC, and BMI with a MDGI value larger than 200.0. Interestingly, TG was the most important health parameter with a MDGI value as high as 459.92 while FPG, WC, BMI, and HDL-C and systolic blood pressure were in the 6 top-ranked informative health parameters as presented in Figure 5. The six significant health parameters were plotted with a 2D scatter plot in Figure 6. The scatter plots displayed MS components that were able to predict MS and non-MS including pairs of TG + FPG, TG + WC, TG + BMI, TG + HDL-C, and TG + BP and other combinations such as FPG + WC, FPG + BMI, FPG + HDL-C, and FPG + BP that were also predictive of MS (Figure 6). The combination of WC + BMI, WC + HDL-C, WC + SBP, BMI + HDL-C, and HDL-C + SBP did not clearly predict MS and non-MS groups, while other combinations could be clearly categorized as MS and non-MS (Figure 6).

In further validating the predictive model, data splitting was performed iteratively for 20 independent runs in order to assess the possibility of chance correlation or overfitting that may have occurred by performing one calculation. Particularly, data splitting of the data set to internal and external sets was followed by the construction of predictive models for the internal set using 10-fold CV as well as assessing the generalizability of the model on the external set. Results from computing the mean and standard deviation of the statistical parameters (e.g., Acc, Sens, Spec, and MCC) from twenty of these independent runs are shown in Table 4. It is clear that our proposed model has successfully predicted MS on the current dataset with accuracies of 97.88 ± 0.18 and 98.12 ± 0.45 as assessed by 10-fold CV and external validation, respectively.

In order to afford practical utility of the obtained predictive model, decision rules were extracted from one of twenty (i.e., the optimal value deduced from empirical optimization) decision tree ensembles of the Random Forest model as shown in Table 5. The relative importance of the decision rules can be implied from the frequency and error imposed by the obtained rules. The most significant rules for classifying individuals as not having MS are those having WC ≤ 79.5 and TG ≤ 150.5.

## 4. Discussion

In this study, the prevalence of MS components in males including WC, TG, FPG, and BP was greater than in females, while the prevalence of HDL-C was higher in women than in men. Other studies have documented the gender-related differences in metabolic abnormalities and in the pattern of lipid abnormalities such as elevated TG in men and low HDL-C in women [30, 31]. Plausible explanation for this could be attributed to conditions such as physical inactivity, dietary behavior, ageing, polycystic ovarian syndrome, and hormonal status. Furthermore, it is noted that premenopausal females tend to develop peripheral adiposity as subcutaneous gluteal fat accumulation whereas men and postmenopausal women tend to have abdominal and visceral obesity [32] that are related to DM and MS. In addition, many studies suggested that excess visceral or abdominal fat were linked to metabolic abnormalities such as insulin resistance and dyslipidemia together with proinflammatory and prothrombotic state [32], which increases the risk of CVD and DM. Hormonal status has been suggested to be involved in MS, particularly as testosterone is converted to estradiol via adipocytes; therefore, the presence of adipocyte cells dysfunction in visceral obesity may influence hormonal abnormalities that may lead to the development and progression of MS [33]. Interestingly, low concentrations of testosterone and sex hormone-binding globulin (SHBG) in men [34] and postmenopausal women (where there is low estrogen levels) [35] have been found to be associated with increased metabolic abnormalities such as visceral obesity, insulin resistance, hyperinsulinemia, and dyslipidemia. Therefore, such differences in gender and basal metabolic states may account for the difference in metabolic abnormalities between males and females.

Identification of MS in an urban adult Thai population was performed using NCEP ATP III criteria that employed the new cutoff for WC, specifically ≥87.75 cm for men and ≥80 cm for women [22], as a component for classifying MS. The prevalence of MS in the adult Thai population studied using NCEP ATP III criteria was 23.70% compared to 21.59% using IDF criteria. Consistent with other studies, we found that the prevalence of MS is also age-dependent and is more common in males than in females [30, 36–38]. However, using NCEP ATP III based on 3 or more metabolic abnormalities, the prevalence of MS in males (34.32%) and females (17.74%) was slightly decreased when compared with previous studies using IDF criteria [30], which was based on WC as the first MS components with 2 or more metabolic abnormalities and was higher, specifically 47.83% and 40.49% in males and females, respectively. Furthermore, WC + BP + TG for males and WC + BP + FPG for females corresponded mostly to metabolic components in a Korean population [15] as well as combination of metabolic abnormalities found in this study. In addition, WBC, Hb, Hct, and PLT were increased in the MS group compared to the non-MS group (Table 1) and have been reported to be associated with insulin resistance and MS [3–7]. Smoking and alcohol consumption were more common in the MS compared to the non-MS group (Table 1). The association between smoking [39–41] and alcohol consumption [42, 43] and MS has previously been reported.

Prediction of MS was performed using RF that exhibited an accuracy greater than 98% for a 10-fold CV and external sets (Table 3) indicating a reliable predictive performance of the model. In previous study, DT, ANN, and SVM have been shown to classify MS with an accuracy of more than 99%, 98%, and 91%, respectively [14]. RF can also classify MS and non-MS as well as DT, ANN, and SVM techniques. RF has been successfully shown to predict MS status based on dietary and genetic parameters with a correct classification rate of 71.7% [44]. Significantly, the Gini index of RF showed that the important variable was the same as what was reported in a previous study using DT analysis [14, 15] and confirmed that TG is the important parameter for predicting MS together with 2 or more metabolic abnormalities. TG is considered to be a significant health parameter that is used as a first screening phenotype characterized with a group of MS components [45, 46]. Furthermore, correlations from previous studies support that TG is the main component that defines MS along with other metabolic abnormalities [15, 16]. Interestingly, doublet MS combinations as shown in Figure 6 are apparently able to predict MS and non-MS. Further, these combinations correlate with previous studies [30, 47] that have explored doublet component combinations of MS, for example, BP + FPG, TG + BP, and TG + FPG that were the most frequent metabolic combinations in males, while BP + FPG, TG + BP, and HDL-C + BP were the most frequent combinations in females [30, 47] that predicted MS status. This result should serve as a guideline for screening individuals who are at risk for developing MS.

The limitation of this study is described as follows: (i) however the class imbalance problem has been documented to affect predictive performance [48], (ii) as the data set was collected from metropolitan Bangkok it may not ideally reflect other regions of Thailand, (iii) results from RF model revealed that TG was the most important MS component, which was not yet verified in this study therefore warranting further validation on its prime importance in the progression of MS. In regard to the first limitation, we did not find that the class imbalance influenced the statistical results in reference to accuracy, sensitivity, specificity, and MCC to predict MS and non-MS (Table 3). As for the second limitation, it can be argued that as the capital of nation there is a high probability that people from all regions migrate to work in Bangkok owing to better job opportunities.

## 5. Conclusion

In conclusion, our findings demonstrated that the RF approach for classifying MS in an adult Thai population has an accuracy of more than 98% and that TG is the most informative variable for the MS component. The important parameters from RF that correlate with the risk of MS based on the NCEP ATP III included TG, SBP and DBP, FPG, and HDL-C. In addition, the prevalence of MS was found to be higher in males than in females and was age-dependent. Therefore, identification of MS using RF holds great utility as a decision support system that could potentially be used for screening MS status, thereby reducing the development of DM and CVD. Practically, the RF approach could potentially be applied in the real clinical setting by applying the RF model on actual data for patients given health check-up.

## Figures and Tables

**Table 1:** Comparison of clinical and biochemical parameters between MS and non-MS groups.

	MS	Non-MS	*P* value
Case number	1,338 (23.70)	4,308 (76.30)	—
Male	696 (34.32)	1,332 (65.68)	—
Female	642 (17.74)	2,976 (82.26)	—
Age (year)	46.99 ± 9.54	40.35 ± 10.41	<0.001
WC (cm)	92.08 ± 9.06	78.52 ± 9.23	<0.001
BMI (kg/m^2^)	27.56 ± 4.21	22.66 ± 3.49	<0.001
SBP (mmHg)	133.28 ± 12.50	119.81 ± 12.62	<0.001
DBP (mmHg)	85.15 ± 9.30	77.47 ± 9.06	<0.001
FPG (mg/dL)	108.35 ± 33.83	90.42 ± 12.73	<0.001
CHOL (mg/dL)	216.63 ± 39.32	205.01 ± 36.00	<0.001
TG (mg/dL)	196.62 ± 90.71	100.36 ± 48.24	<0.001
LDL-C (mg/dL)	131.21 ± 46.98	121.23 ± 32.68	<0.001
HDL-C (mg/dL)	48.07 ± 11.19	63.78 ± 14.48	<0.001
WBC (×10^9^/L)	7.26 ± 1.77	6.46 ± 1.56	<0.001
Hb (g/dL)	14.28 ± 1.55	13.60 ± 1.47	<0.001
Hct (%)	41.97 ± 4.19	39.92 ± 3.98	<0.001
PLT (×10^9^/L)	274.38 ± 66.21	261.71 ± 60.70	<0.001
Smoking	0.105 ± 0.307	0.057 ± 0.232	<0.001
Alcohol	0.372 ± 0.484	0.296 ± 0.457	<0.001

Data were expressed as the mean ± SD or as percentages. MS: metabolic syndrome, non-MS: nonmetabolic syndrome, WC: waist circumference, BMI: body mass index, SBP: systolic blood pressure, DBP: diastolic blood pressure, FPG: fasting plasma glucose, CHOL: total cholesterol, TG: triglyceride, LDL-C: low-density lipoprotein cholesterol, HDL-C: high-density lipoprotein cholesterol, WBC: white blood cells, Hb: hemoglobin, Hct: hematocrit, and PLT: platelet. Smoking and alcohol refer to individuals who smoke cigarettes and consume alcohol.

**Table 2:** The number of subjects used as internal and external validation sets for predicting MS.

Status	Initial	Internal validation set	External validation set
MS	1337	1137	200
Non-MS	4306	3659	647
Total	5643	4796	847

**Table 3:** Summary of statistical parameters for MS classification using Random Forest.

*n*_tree_	Internal test set (10-fold CV)				External test set			
	Acc	Sens	Spec	MCC	Acc	Sens	Spec	MCC
10	97.10	94.28	97.98	0.92	97.99	95.00	98.92	0.94
20	97.94	95.07	98.82	0.94	98.11	94.00	99.38	0.95
30	98.02	94.72	99.04	0.94	97.64	92.00	99.38	0.93
40	98.02	94.81	99.02	0.94	97.76	92.50	99.38	0.94
50	98.02	94.64	99.07	0.94	97.76	92.50	99.38	0.94

10-fold CV: 10-fold cross-validation, Acc: accuracy, Sens: sensitivity, Spec: specificity, and MCC: Matthews correlation coefficient.

**Table 4:** Summary of prediction performance for MS classification using Random Forest from 20 independent runs.

Prediction performance	Internal test set (10-fold CV)				External test set			
	Acc	Sens	Spec	MCC	Acc	Sens	Spec	MCC
Mean	97.88	94.54	98.91	0.94	98.12	94.80	99.15	0.95
SD	0.18	0.65	0.12	0.00	0.45	1.49	0.45	0.01

10-fold CV: 10-fold cross-validation, Acc: accuracy, Sens: sensitivity, Spec: specificity, and MCC: Matthews correlation coefficient.

**Table 5:** Decision rules extracted from one of twenty trees from the predictive model trained with Random Forest.

Frequency (%)	Error (%)	Condition	Prediction
42	0	WC ≤ 79.75 and TG ≤ 150.5	Non-MS
10.1	0	FPG ≤ 99.5 and TG ≤ 149.5 and HDL-C > 49.15 and LDL-C ≤ 128.05	Non-MS
9.6	0	WC > 87.75 and systolic > 127 and TG > 149.5	MS
3.3	0	Sex = female and WC > 79.5 and systolic > 125 and FPG > 99.5	MS
1.7	0	WC > 87.5 and systolic > 128 and FPG > 99.5	MS
1.7	0	Sex = female and WC > 80 and TG > 150.5 and HDL-C ≤ 48.1	MS
1.7	0	WC ≤ 87.75 and systolic ≤ 127 and Hct > 44.77	Non-MS
1.3	0	Sex = female and WC > 79.5 and systolic > 129 and HDL-C ≤ 49.75	MS
8.7	0	FPG ≤ 99.5 and TG ≤ 149.5 and HDL-C > 49.15	Non-MS
1.1	0	FPG ≤ 99.5 and TG ≤ 149.5 and HDL-C > 38.7 and Hct > 44.1	Non-MS
1.1	0	Systolic > 125 and FPG > 99.5 and TG > 149.5	MS
1.5	0	WC ≤ 78.5 and HDL-C > 50.45	Non-MS
1	0	WC > 87.75 and FPG > 99.5 and TG > 148.5	MS
1.5	1.1	WC ≤ 87.5 and TG ≤ 150.5 and Hct > 42.305	Non-MS
1.8	1.9	FPG ≤ 99.5 and TG ≤ 149.5 and HDL-C > 39.6	Non-MS
2.1	2.5	Systolic ≤ 125 and diastolic ≤ 85 and TG ≤ 149.5 and HDL-C > 47.2	Non-MS
2.5	2.9	Sex = male and WC ≤ 87.75 and HDL-C > 39.95	Non-MS
1	6.8	Diastolic > 85 and TG > 149.5	MS
1.6	6.5	WC ≤ 87.75 and systolic ≤ 127 and FPG ≤ 99.5	Non-MS
1.1	0	TG > 150.5 and HDL-C ≤ 40.05	MS
